# Balancing different expectations in ethically difficult situations while providing community home health care services: a focused ethnographic approach

**DOI:** 10.1186/s12877-018-0996-8

**Published:** 2018-12-14

**Authors:** Dara Rasoal, Annica Kihlgren, Kirsti Skovdahl

**Affiliations:** 1 School of Health, Care and Social Welfare, Mälardalen University, Högskoleplan 1, 721 23, Västerås, Sweden; 2 Institution of Health and Medical Sciences, Örebro University, Faultetsgatan 1, 701 82 Örebro, Sweden; 3 Faculty for Health and Social Sciences, University in South-Eastern Norway, Post box 235, 3603 Kongsberg, Norway

**Keywords:** Ethically difficult situations, Community home health care services, Healthcare professional, Ethnography

## Abstract

**Background:**

The general opinion in society is that everyone has the right to live in their own home as long as possible. Provision of community home health care services is therefore increasingly common. Healthcare personnel encounter ethically difficult situations when providing care, but few studies describe such situations in the context of community home health care services.

**Method:**

This study has a qualitative descriptive design, using focused ethnography. Data from 21 days of fieldwork (in total 123 h) consisting of non-participant observations (*n* = 122), memos and informal interviews with registered nurses (*n* = 8), and nurse assistants (*n* = 4). The transcribed texts were analyzed with interpretive content analysis.

**Results:**

The inductive analyses revealed two categories: 1) difficulties in balancing different requirements, expectations and needs, and 2) use of coping strategies. The results demonstrate that there are different values and expectations that influence each other in a complex manner. The personnel dealt with these situations by generating strategies of coaxing the patients and finding a space to deliberate and share difficult emotions with their colleagues.

**Conclusions:**

This study reveals that complex ethically difficult situations emerged in the context of community home health care services, and healthcare personnel were forced to find a balance regarding the different demands, expectations, values and needs that influence the care provided.

## Background

In recent decades there has been a significant rise in the number of people who receive community home health care services. The general opinion in society is that everyone has the right to live in their own home as long as possible [1, 2]. One purpose of living at home and receiving home health care is to maintain and enhance a person’s quality of life [3]. Another is to assist people to live independently and to achieve a life as normal as possible despite health-related issues and demands. The way community home health care services function with respect to the care provided varies among European countries [4]. In Norway, municipalities have responsibility for social care; while provision of some long-term care services is statutory, budgets are set locally [5]. The Norwegian welfare model is publically funded and is relatively well resourced by international standards. A priority is delivering care in line with the principles of justice and ensuring access to quality care regardless of the patient’s background or economic status [5, 6]. In addition, health care institutions emphasize that care provision should be aligned with the concept of person-centred care [7–9]. Person-centred care highlights the importance of knowing the person behind the patient as an individual with reason, free will, feelings, and needs, of considering the person’s context and of engaging the person as an active partner in his/her care and treatment [10, 11]. In addition, the care should be provided to as many patients as possible in an efficient and economical way. Community home health care services offer care that is administered at home by certified healthcare personnel such as registered nurses and nurse assistants. Community home health care services provide: a) post-acute recovery care, b) maintenance of functionality care for older people who need support to remain at home for as long as possible with a good quality of life, c) palliative care [12, 13], reablement and rehabilitation [14] and/or daily assessments and care for patients with psychiatric disorders [15].

Delivery of complex, essential and advanced home health care is likely to generate challenges in terms of managing the ethical aspects of care. Previous research has reported how healthcare personnel working in community home health care services face ethically difficult situations on a daily basis [16, 17]. Ethically difficult situations occur when personnel encounter situations in which they are uncertain of which values and whose values should or could be applied [18–20]. The consequences of feeling uncertain or insecure regarding what care to provide can cause a sense of powerlessness [21] and feeling insufficient can lead to moral distress. Previous work has described how institutional constraints [22, 23], inadequate resources in the form of a personnel shortages, lack of respect from patients and managers [24], problems with consent for treatment [25], and powerlessness over work situations can also impact on the personnel’s feelings [26]. Ethically difficult situations have been described in the context of hospitals [21, 27–29] as well as in nursing homes when providing care for older people [18, 30–32]. These situations have been examined among healthcare personnel using face-to-face interviews [30], focus groups [33], questionnaires [28, 34] and telephone surveys [35].

However, to our knowledge there is a lack of studies describing ethically difficult situations using an ethnographic method in which personnel are observed in clinical practice and are informally interviewed. This study aims to describe the healthcare professionals’ actions and experiences of ethically difficult situations while providing community home healthcare services, and how they deal with these situations.

## Method

The study applied a qualitative descriptive design, using focused ethnography [36, 37]. This approach is used in order to describe a specific culture, in this paper, community home health care services, and the meanings of actions and the events that occur within that culture [38]. This approach is appropriate for studying a group of people who share a common experience [37, 38], for example, ethically difficult situations in their everyday work in health care [39]. Through a focused ethnographic approach the researcher obtains an emic perspective, i.e., an *insider* view of a social group’s experiences, as well as an etic perspective or *outsider’s* view [40], i.e., an interpretation of what the participants experience in relation to the observed situation from someone outside the social group [36].

### Setting and participants

This project was initiated by a manager in a community home health care services district in a medium large municipality in Norway, who contacted a university research group for collaboration. The manager acted as a ‘gatekeeper’ for this study. All of the certified personnel (*n* = 26: registered nurses *n* = 10 and nursing assistants *n* = 16) in that district were invited to a staff meeting where two of the authors and the manager informed them about the study. The authors asked them to read the written information and decide if they were willing to participate. The first author returned after a couple of days and provided more detailed information to personnel who were willing to participate. Inclusion criteria for participation were personnel who were certified and ordinarily worked in the setting. Staff members who worked for a private company or worked only a few shifts were excluded. A total of eight registered nurses and four nurse assistants were included in the study. They were males (*n* = 2) and females (*n* = 10), aged between 20 and 58 years (mean = 41) with from one to 20 years (mean = 12) of experience working in health care.

### Description of healthcare professionals’ daily work routines

The nurses and nurse assistants started every shift at the community home health care services office, where they received an oral report from their colleagues on the previous shift. This was followed up by reading the documentation in the patient files. This became the basis for how they planned their shifts and deciding which patients they needed to prioritize. Generally, patients who needed immediate care or medication were prioritized before patients with less acute needs. During the day shift, each member of the team (registered nurse or nurse assistant) was responsible for approximately 20 patients with various health care needs. The distance to the patients’ homes from the office varied between 200 m to 10 km. Most of the patients observed in this study were living in individual apartments in multi-story buildings with a single bedroom, kitchen, bathroom and living room. The staff member entered the patient’s home with a key obtained from a secure box outside the door. Before entering, they usually rang the doorbell to announce their arrival. The services/actions provided were support and basic care to persons who had various psychiatric, cognitive or somatic disorders, and functional disabilities. The services included bathing, dressing, meal preparation/feeding, giving medication, changing bandages, transferring and lifting.

### Data collection

Data were collected in April and May 2016 through repeated non-participant observations and informal conversations [36], during the day shift between (08:00–16:00) and in the evening between (16,00–22:00). Each day of field work lasted from five to seven hours and comprised 1–6 visits at each patient’s home. The observations (*n* = 122) were conducted over three weeks’ time in different patients’ homes (*n* = 22) and the total observation time was 123 h. Field notes of the observed interactions were written down, during and after each observation. In relation to the observed situations, the researcher asked the staff member questions related to the observed situations to obtain their perspective. The questions included “how did you experience this situation?” or “was there anything you experienced as ethically difficult in that situation?” or “could you tell me more about that?”. In this way, the nurses and nurse assistants were able to describe their experiences of situations they perceived as problematic, and were given opportunities to draw out ethical aspects of the situations they experienced.

In addition, informal conversations with personnel were conducted after returning to the community home health care services office to further illuminate various aspects of the ongoing interactions and to obtain a deeper understanding of the meanings of the actions. Notes were written during these conversations which lasted between ten minutes and one hour. At the end the researcher summarised the data by recording his impressions on a dictation device. Since the observations were repeated, the researcher was provided with opportunities to discuss what had happened during prior observations with personnel on subsequent occasions.

### Data analysis

All data, field notes, observations and recorded data, were transcribed and organised as a whole into the software Nvivo10 [41]. The texts were analyzed with interpretative content analysis [42]. The first author (DR) read the material several times aiming to grasp the meaning in the texts. Guided by the aim of the study, the meaning units were identified and condensed into a description close to the text in order to catch the manifest content. These condensed meaning units were abstracted and coded. Through continuous comparisons of similarities and differences, the codes were grouped into subcategories. The next step was a reflective interpretative process, which was a back-and-forth process between meaning units, codes, and subcategories as threads of meaning running through them. The research group discussed the analyses and possible interpretations of the result until a consensus was reached. Two categories emerged: 1) *difficulties in balancing different requirements, expectations and needs* and 2) *use of coping strategies,* which together led to the emergence of seven subcategories. Throughout the analysis process, the researchers scrutinized the results to ensure the credibility of the analysis.

### Ethical considerations

Approval for the study was obtained from the Norwegian Social Science Data Services (NSD) (ref. 47,995) and Regional Research Ethics Committee (ref. 2016/673). Oral and written information regarding the study aim was provided to all personnel and the patients in advance. The information letter clarified that participation was voluntary, and that they could withdraw their participation at any time without any consequences for none of them. All personnel (*n* = 12) and patients (*n* = 22) gave their oral and written consent to participate in this study.

The researcher was took care to be aware throughout the period of observation whether there were any signs from the patient that he or she was disturbed by the researcher and to dismiss himself if this was the case. The participants were promised that the collected data would be protected and confidentiality was guaranteed in accordance with research ethics and law [43, 44]. The quotations used in the results section are slightly modified in order to reduce the risk of participants being recognised.

## Results

**Fig. 1:**
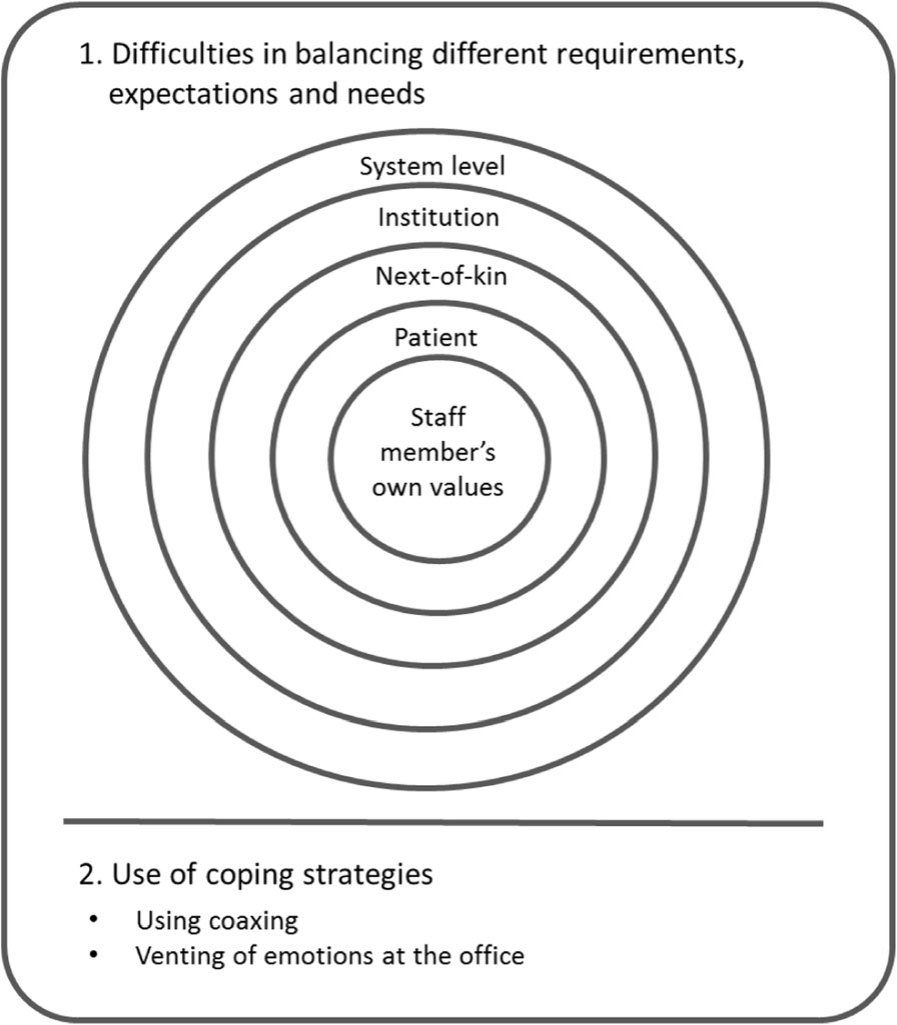
Overview of categories and subcategories emerging from analyses of ethically difficult situations occurring in the course of provision of community home health care services in a Norwegian municipality

Through an inductive approach in which all researchers participated, we identified seven subcategories (Fig. 1). Five of these related to factors that influenced the provision of care. These existed at a variety of levels, from staff members’ own values, interactions with patient and next-of-kin, and constraints on delivery of care from their workplace as well as the national context. We grouped these five subcategories into a first category called *difficulties in balancing different requirements, expectations and needs.* This category contains five subcategories, which can be understood as competing demands at different levels: 1) *difficulties to fulfil the requirements from a system level*, 2) *organizational factors impeding the provision of care,* 3) *difficulty meeting needs of next-of-kin*, 4) *difficulty meeting patients’ needs, 5) uncertainty how to manage personal values in caregiving situations.* The remaining two subcategories described staff behaviour in response to these competing pressures. We classified these into the second category *use of coping strategies*, with the subcategories: 1) *using coaxing as a strategy in order to deal with difficult situations*, and 2) *using the office as an arena for deliberation and venting of emotions.* The relationships between these categories and subcategories is displayed in the figure.

Although the focus of the inquiry was on ethically difficult situations, participants talked about difficulties more broadly, without always invoking their potential ethical aspects. Therefore, in the first category, the multiple pressures staff reported experiencing generated situations which were viewed as problematic, and which we were able to observe could generate value-laden conflicts and a sense of unease. In the second category, concerning coping strategies, these strategies in themselves could raise ethical concerns, and did at times among participants, particularly concerning the use of the office to vent emotions.

### Difficulties in balancing different requirements, expectations and needs

#### Difficulties to fulfil the requirements from a system level

The law combined with the ideology of person-centred care were important factors that could be experienced by personnel as demanding in terms of providing care that was safe, high quality, equal, and individualised. Considering the patient’s self-determination and providing care according to their wishes could generate situations that sometimes were experienced as being in conflict with demands to provide safe and equal care.

*“it is really difficult [….]…we have all the pressure from society, law and from the county [….] and everything goes quicker. How can we provide dignified care when you have all this pressure on you [….] Everyone talks about how the older people should be treated and so on [….] when I was at school we read all these books talking about how the patient should be treated, but when you start to work, you see that is different.”* (Informant 1)

Staff described a kind of implicit pressure that occurs as a result of obligations in their everyday work. They experienced difficulties fulfilling their professional obligations and providing care in accordance with ethical codes.

#### Organizational factors impeding the provision of care

The personnel described that they followed a set routine provided by the organisation in planning care for the different patients. They experienced pressure from the home health care organisation to provide the necessary care in a rather limited amount of time, which was further exacerbated by what was perceived to be a far too large number of patients. Repeated observations showed that provision of care seemed to be complex and influenced by various interests.

One of the staff members came to the patient’s home and started with the regular preparations for washing the patient, preparing food and dressing the patient. The patient was happy to have the staff member in their house and said she had been waiting and started to talk about her life. The staff member nodded, continued working and seemed to be little stressed after 15 minutes and looking at her watch said “*Oh the time flies*” and started to speed up the care being given by leaving the patient alone in the bathroom to dress herself and went to the kitchen preparing some breakfast as well as medications. […] The staff member wanted to leave the patient, but the patient pleaded with her not to go. The staff member said that she had to because others are waiting and left. Observation

The researcher observed that the personnel carried with them a device that showed how many patients they had to provide care to within a certain time. They hurried to different homes in order to provide care. It was also observed several times that they rushed by driving the service car over the speed limit. In addition, personnel described difficulties finding a way to provide sufficient care without impinging on anyone’s values or interests:

*“...you ask me what is difficult…well I mean of course...it’s like… it is difficult when you cannot deliver care in accordance with patients’ wishes or requests… all you need is just to deliver… and the fact is that the patient cannot always get what they want…but we do what we can….it is a matter of time and resources... you don’t have the capacity, which is not good”* (Informant 6)

Personnel needed to consider the patient’s wishes and interests in the different caregiving situations and it was their intention to involve patients. Some patients needed more care, time and attention from staff members than was planned and allocated for from an organisational level.

#### Difficulty meeting needs of next-of-kin

The personnel felt that next-of-kin could cause difficult situations when they demanded more medication for their relative who were in pain and the staff member felt such demands from next-of-kin were inconvenient. The nurses explained that they were trying to do their best for the patients, to help them feel better and ease their pain, but that next-of-kin hindered them from doing their job:

The patient’s mother was standing in front of the gate and seemed to expect the personnel’s arrival. The mother started to cry, and said her son was in pain and needed some more medication and asked the nurse to give it to him. The nurse answered that she did not have the authority to do so, and was not allowed. The mother continued to cry and blamed them for not doing the right thing. The mother said “How can you treat a human like that, this is not compassion.” The nurse said: “If I give extra medication without an order from the physician, I could lose my job and I can’t do that.” Observation

Another observation showed that an older person did not want to eat breakfast for some unknown reason, but the personnel convinced the person to eat her breakfast. The personnel experienced situations where they were directed by the next-of-kin to feed the patient, even though the patient did not want to eat. One staff member expressed her worries over this kind of situation and how it can be experienced as problematic:

*“[….] you could get into trouble if you didn’t make sure the patient eats. It happens that they (next-of-kin) complain to you, for example they tell us you are not doing your job or you must make sure that daddy is eating, and that is problematic at times.”* (Informant 10)

The observation revealed that ethically difficult situations could also arise regarding the information that concerns the health conditions of a patient. Next-of-kin often called and asked personnel to update them concerning the health condition of their parents. Nurses and nursing assistants have asked patients if they can give out sensitive information concerning patients’ health conditions to next-of kin, and sometimes the patients declined. Personnel perceived such situations as ethically demanding. A conflict could develop between them and the next-of-kin if personnel respected the patients’ wishes and did not provide any information to next-of-kin. If personnel disclosed any information to the next-of-kin, they would be in conflict with the patient and the law and break the rule of confidentiality.

*“….They want us to inform them about everything we do for the patient, and sometimes they [the patient] don’t want us to give them the information and we try to inform the next-of-kin about that, but they have difficulty accepting that. It can give rise to conflict.”* (Informant 2)

#### Difficulty meeting patients’ needs

The observation revealed that conflicts could occur between personnel and the persons who received healthcare. One type of conflict concerned disagreements on which medication to take where the patient acted emotionally and showed anger which resulted in speaking unkindly to personnel. Personnel described that they felt the patients were not co-operating with them on what was considered the ‘best course of action’ related to the use of drugs. This led to frustration among personnel and they described that these situations were ethically difficult to handle. They tried to create good days for the patients and recreate a good relationship and trust in order to help them reduce their use of drugs. Consequently, care was not provided in the way the patient wanted, but according to what personnel thought was best for the patient:

One staff member entered a patient’s house, provided a new narcotic-based medication and told the patient that that the doctor had decided to reduce the dosage. The staff member also informed the patient about the new type of medication. The patient became angry and refused to accept that. The staff member said that they understood the patient’s feelings and would report back to the physician. Observation

According to the personnel, situations like this started when they trusted the patient in the beginning and an agreement had been reached as to how the narcotic-based medication would be tapered down. Unfortunately, when the patient (according to personnel) failed to abide by the agreed contract, the patient was perceived to be irresponsible by personnel.

The personnel seemed to be frustrated and angry with such situations especially when patients had children at home who could see their parent using strong narcotic-based medication and its effects. If they responded to patients’ pain and provided more medication, personnel expressed that they felt they were somewhat responsible and had contributed to patients’ addictions when the dosage was increased instead of decreased:

*“It is very difficult because … we feel in some way we are helping to keep a person high on drugs all the time. This patient gets a lot of medication. It is difficult to know what is ethically right in this particular situation, especially when we have to deal with someone we don’t know, if the patient is psychologically addicted to this medication or suffers from an illness [….]”* (Informant 9)

#### Uncertainty about how to manage personal values in caregiving situations

The observations showed that personnel seemed to be affected by their own values or the values of their colleagues on what the best course of action was in different situations. Time and effectiveness seemed to be prioritized before patients’ needs, desires and involvement in the caregiving situations. One observation showed that personnel in many situations made decisions without consultation or collaboration with the patient. Another observation showed that personnel provided the most basic care and spent less time with some patients. Additionally, it was observed that staff members assumed differing attitudes and behaviours when entering patients’ homes. In some homes, they tended to take off their shoes, while in others they continued to wear them. The researcher asked a staff member why they took off their shoes in some homes but not in others and received the response:

*“It is dirty anyway there…and I don’t want dirt on my feet. And it also not polite to carry dirt from that place to another patient’s home that is cleaner”* (Informant 1)

Another repeated observation revealed that personnel seemed to delay their response to some patients who were in pain. Some personnel had concluded that the patient’s pain was not legitimate, a conclusion partly based on what colleagues had said during informal meetings and conversations. Others expressed that they had a sense of guilt when they did not provide equal and justified care to all patients:

A patient rang on the telephone and asked the nurse for a painkiller. The personnel (nurses) started to discuss with the patient who, according to the nurses, often calls and requires a strong painkiller for his/her stomach. […] The staff members were divided about how best to respond to this patient. Some of them doubted the patient’s pain and did not deliver painkillers immediately, but asked the patient to wait. Another nurse said: we should respond to this patient exactly in the same way as the other one since both have the same disease, and referred to doctor’s order. We should treat them equally and in exactly the same way, but we don’t. Observation

### Use of coping strategies

The observations also revealed how healthcare personnel employed coping strategies in order to attempt to balance their own interests with the interests of patient, next-of-kin and the home health care service as a whole.

#### Using coaxing as a strategy in order to deal with difficult situations

The nurses and nurse assistants had developed a strategy they called “coaxing” (in Norwegian Bokmål, “lirke”) as a strategy to deal with difficult situations. We were able to understand “coaxing” through observation and participant explanations as efforts by staff members to get patients to comply smoothly with their wishes. Several observations showed that they were manoeuvring the conversation with the patients towards particular goals, especially when the patients often refused food or showering. In addition, they tried to involve the patient by using what seemed to be a kind-hearted attitude mixed with the coaxing. In this way, it seemed that the patient felt they were involved and had the impression that the suggested choice was their own rather than the personnel’s. We view such interactions as ethically problematic, due to the asymmetrical power relationship on view here. However, the staff members described this coaxing strategy as an effective way to deal with difficult situations:

*“So the question is if it is ok to coax the patient? Sometimes if you let them think what they want and how they want it, there is a risk that you’ll never get an answer.”* (Informant 4)

According to personnel, the strategy of coaxing the patient seemed to work, especially with older patients. However, they explained that they must respect the patient’s wishes if they were not hungry, but at the same time they found it difficult to understand why the patient did not want to eat. Personnel explained that they did not have much time to spend with each patient and found coaxing to not only be an effective, but also efficient method of pushing the patients to get up, do their morning rituals and get something to eat. This can be exemplified with this observation:

The staff member took the initiative and asked the person to get up and eat. The person said: *“I don’t want to eat”.* The staff member coaxed the person for a few minutes in an attempt to persuade the person to eat before the staff member gave up and put the food on a table or in a refrigerator. Observation

The nurses and nurse assistants explained that they had other patients waiting to be taken care of and coaxing was used as an acceptable method to give the patient the best possible care and save time**.** Even though coaxing was employed as a means to achieve something good for the patient, some staff members did express concerns that this approach was not based on patient self-determination and choice.

#### Using the office as an arena for deliberation and venting of emotions

The observations showed that another way to deal with ethically difficult situations was to express negative emotions in the office of the community home health care service. While personnel expressed the need and desire to use the office as an arena for reflections and exchange of new ideas on how to provide good care, it had instead become an arena for expressing frustrations and feelings about certain patients. Such frustrations appeared to result from difficulties staff members faced in trying to support certain patients with their medical issues. Staff members expressed a desire that the office could instead be used for reflection and for the exchange of new ideas about how to provide good care:

*“I think that the office is an arena where one can reflect and ventilate thoughts. It is important I think, but to say bad things about a patient is nothing more than bad talk, no doubt about that. A lot of what we do is unprofessional [….] I don’t want to make a complaint here, but to speak badly about another is not good”.* (Informant 10)

Speaking in negative terms about certain patients was observed. This seemed to be a way of problematizing and clarifying aspects that they were struggling with, both ethically and emotionally. It was observed that personnel spoke openly in an unkind manner about the patients by labelling them different things such as “children,” and “demanding” when certain patients needed more care or attention. This was observed only when managers were not present. Personnel felt they needed these opportunities to ventilate their feelings so they could move on and continue with their everyday work.

## Discussion

The aims of this study were to describe healthcare personnel’s actions and experiences of ethically difficult situations while providing community home healthcare services, and how these situations were dealt with by the personnel. The results show that despite the fact that much care provided was basic, complex ethical care situations emerged due to several factors. Factors which seem to affect the care in this study were based on the prerequisites and conditions for how care should be provided. These needs were based on a top-down approach which began at the system level and continued down to through the organization to the patient, next-of-kin and staff level. The personnel seemed to be forced to find a balance regarding different expectations, values and needs from the system to the organization, and from the organizational level to the patients and next-of-kin, on personal levels that were influencing the care provided. There are demands from the law [45, 46], the next-of-kin [47, 48] and patients themselves that the care must be provided and delivered in a prescribed manner. The demands from the system level are legal obligations to deliver health care in accordance with certain rules and codes of ethics, i.e., to provide equal, dignified and good care and to consider the patient’s self-determination in the care situation [43, 44, 49, 50]. These demands seemed to be difficult to fulfil and staff took issue with them due to the lack of resources.

Other challenges were the interests and expectations from the next-of-kin that were not in accordance with the wishes of the patient, nor recommendations from the personnel. Next-of-kin have been described in several previous studies as having power in the caregiving process as well as unrealistic expectations [34, 51] In addition, personnel can feel powerlessness when trying to manage difficult interactions with patients and next-of-kin [52].

The recommendations made by personnel were not always in accordance with the wishes of the patient, and patients sometimes showed mistrust for the personnel [27, 53]. It seemed that loss of trust had occurred between personnel and patients. Personnel seemed to have difficulties recreating the trust they had with some patients that they had from the beginning. Personnel described their previous experiences from the situations they had been in with particular patients as reasons for the loss of trust. The personnel’s disappointment was based on the detection that patients had failed to comply with requests to taper down medication. This is in line with previous studies describing disagreement and divergent points of view among healthcare personnel [54–56]. Personnel seemed to have divergent views on what was ‘good care’. Several of them did not want to provide care in the same way as their colleagues. The pressure from the group was however bigger than individual staff members could handle emotionally. Personnel with long work experience and more qualifications seemed to have a more dominant voice in the group [52]. A possible interpretation is that personnel did not dare to voice their disagreements in front of their colleagues in the office. These opposing expectations and values mostly concerned issues regarding medication. However, these values and expectations that influenced the care were not constant, but varied depending on the situation, the staff member providing care and the individual patient.

The nurses and nurse assistants seemed to have developed coaxing as a strategy to deal with difficult care situations. Even though they used coaxing as a strategy to achieve their goals in providing care, they expressed uncertainty regarding whether coaxing was in fact the best course of action. Rather than being a negotiation between two equal parties, this strategy is effective due to a power asymmetry in which the staff member takes the initiative and attempts to persuade to do what the staff member perceives as the best course of action. In addition, they shared their frustrations over the shortcomings concerning these care situations with each other. Disappointment over such situations could generate feelings of frustration. The consequences of these frustrations could result in the personnel saying unkind things about patients, e.g., labelling the patient as being as ‘difficult’ or ‘demanding’ [27]. According to Jeffery [57] and Dingwall [58], it is not uncommon that healthcare personnel label patients as being ‘difficult’ and speak negatively about them. An important aspect occurred when a person was treated as a ‘diagnosis’ more than as a person with values and self-determination. These kinds of attitudes or situations place the values of person-centred care at risk. According to the values of person-centred care, the person, in her/his context and in terms of how their health-related demands affect their daily lives, has the right to be involved in their care process as much as possible without being undermined [7, 11, 59]. In order to provide care founded on person-centred care values, it is crucial to have a mutual relationship that is built on respect in which personnel are aware of the patient’s personal values and wishes [60].

The potential for building a relationship within the context of community home health care services may differ in comparison with that of institutional care. In home health care the relationships are often long-term, which provides opportunities for the development of a flourishing relationship [60]. This may merely be an ideal, since our results showed that the personnel were struggling with lack of time and pressure to deliver effective care and help as many patients as possible during their shift. Lack of time and other prerequisites may have influenced and limited the options for building relationships. In order to achieve the values of person-centred care at an individual level, prerequisites such as time, competencies, and support at an organisational level are crucial [60]. In addition, structures to support ethical reflection may be useful to enhance the staff members’ awareness of the complex ethical situations arising in their everyday work, and offer support for the development of tools to deal with them in the context of home health care.

### Strengths and limitations

The repeated observations and staff members’ experiences gave rich and ample data which we see as a strength in this study. Another strength is that the project was initiated by the community home healthcare personnel service which motivated them to provide rich and detailed data concerning their everyday experiences and their needs. Questions to staff members were open and related to the situations occurring during the observations. *A possible* limitation might be that the staff members may have acted somewhat differently in the presence of the researcher, but since the observations were performed with different personnel and were repeated, this limitation may have been reduced.

All authors have previous experience of analysing ethical and other issues in healthcare, and this knowledge will have informed both data collection and analysis. The first author tried to be as open-minded as possible when observing actions and tried to be aware of any personal pre-understandings that could affect the field notes. Having a pre-understanding and being a part of the field can unavoidably impact the result, but at the same time pre-understanding can help the researcher to see new aspects of the reality [61, 62]. To strengthen the trustworthiness of the result, member checking [63] was conducted with personnel and a constantly critical dialogue was held with a researcher who is familiar with the field, but who was not involved in data collection.

## Conclusions

Despite the fact that most care provided during the observed situations was basic (e.g., showering, providing medication and food), these caring situations generated highly complex ethical situations. Home health care personnel were forced to find a balance regarding different demands, expectations, values and needs from the system, organisation, and on the personal level. These levels had a profound impact on care and created various ethically difficult situations in the provision of care. One way to deal with these situations was through coaxing and another way was through sharing their frustrations with each other. However, this latter strategy sometimes resulted in personnel saying unkind things about patients, e.g., labelling them as ‘difficult’ or ‘demanding’ which challenges the idea of dignified care and patient self-determination. This study provides a new aspect regarding the recognition of ethical challenges in community home health care service and a method of dealing with them: coaxing, a strategy which may raise concerns for patient autonomy.

The results of this study may be transferable to similar contexts, but further studies are needed to verify this, since the current study took place in only one district in the Norwegian community home health care setting. In addition, future studies are needed to complement these observations by focusing on patient perspectives and their experiences of receiving care at home.

In terms of community home health care practice, introducing ethics support in the form of collective systematic reflection in the form of ethical rounds or ethical discussion groups could be beneficial [64]. These initiatives may help support healthcare workers to develop greater awareness of the ethical aspects of providing care and may support healthcare workers in approaching ethically difficult situations.

## References

[1] Henderson EJ, Caplan GA (2008). Home sweet home? Community Care for Older People in Australia. J Am Med Dir Assoc.

[2] Mentsen T, Hellzen O, Enmarker I (2015). The Experience of Nurses Providing Home Nursing Care to Oldest Old Persons Living Alone in Rural Areas. An Interview Study.

[3] Low L-F, Yap M, Brodaty H (2011). A systematic review of different models of home and community care services for older persons. BMC Health Serv Res.

[4] Genet N, Boerma W, Kroneman M, Hutchinson A, Saltman R. Home care across Europe: case studies. Utrecht, The Netherlands: European observatory on Health System and Policies; 2013. https://www.narcis.nl/publication/RecordID/publicat%3A1002336. Accessed 11 Nov 2016.

[5] Lindahl AK. Norway: Int Health Care System Profiles. The Commonwealth Fund. n.d. https://international.commonwealthfund.org/countries/norway/. Accessed 27 Oct 2018.

[6] HOD H 1999. Patient and Service User Law. HOD 1999-07-02-63. Lov om pasient- og brukerrettighet. Oslo. p. 1999. https://lovdata.no/dokument/NL/lov/1999-07-02-63

[7] Ekman I (2014). Personcentrering inom hälso-och sjukvård: från filosofi till praktik.

[8] Ekman I, Hedman H, Swedberg K, Wallengren C (2015). Commentary: Swedish initiative on person centred care. BMJ.

[9] Sandman L, Granger BB, Ekman I, Munthe C (2012). Adherence, shared decision-making and patient autonomy. Med Health Care Philos.

[10] Mounier E (2013). Personalism.

[11] Ekman I, Swedberg K, Taft C, Lindseth A, Norberg A, Brink E (2011). Person-centered care — ready for prime time. Eur J Cardiovasc Nurs.

[12] Tousignant M, Dubuc N, Hébert R, Coulombe C (2007). Home-care programmes for older adults with disabilities in Canada: how can we assess the adequacy of services provided compared with the needs of users?. Health Soc Care Community.

[13] Parks JA (2003). No place like home?: feminist ethics and home health care.

[14] Sims-Gould J, Tong CE, Wallis-Mayer L, Ashe MC (2017). Reablement, reactivation, rehabilitation and restorative interventions with older adults in receipt of home care: a systematic review. J Am Med Dir Assoc.

[15] Jokstad K, Landmark BT, Hauge S, Skovdahl K-I (2016). Eldres erfaringer med hverdagsrehabilitering - Mestring og muligheter − krav og støtte i et dynamisk samspill. Tidsskr Omsorgsforskning.

[16] Karlsson M, Karlsson C, Barbosa da Silva A, Berggren I, Söderlund M (2013). Community nurses’ experiences of ethical problems in end-of-life care in the patient’s own home. Scand J Caring Sci.

[17] Anstey KW, Wagner F. Community healthcare Ethics Camb Textb Bioeth. 2002;:299.

[18] Slettebø Å, Bunch EH (2004). Solving ethically difficult care situations in nursing homes. Nurs Ethics.

[19] Altun I (2002). Burnout and nurses’ personal and professional values. Nurs Ethics.

[20] Bentzen G, Harsvik A, Brinchmann BS (2013). Values that vanish into thin air: nurses’ experience of ethical values in their daily work. Nurs Res Pract.

[21] Grönlund CF. Doctoral thesis / doktorsavhandling. In: Experiences of being in ethically difficult situations and an intervention with clinical ethics support. Umeå University; 2016.

[22] Jameton A (1984). Nursing practice: the ethical issues.

[23] Erlen JA (2001). Moral distress: a pervasive problem. Orthop Nurs.

[24] Maluwa VM, Andre J, Ndebele P, Chilemba E (2012). Moral distress in nursing practice in Malawi. Nurs Ethics.

[25] Austin W, Kelecevic J, Goble E, Mekechuk J (2009). An overview of moral distress and the paediatric intensive care team. Nurs Ethics.

[26] Ulrich C, O’Donnell P, Taylor C, Farrar A, Danis M, Grady C (2007). Ethical climate, ethics stress, and the job satisfaction of nurses and social workers in the United States. Soc Sci Med.

[27] Hermsen M, van der Donk M (2009). Nurses’ moral problems in dialysis. Nurs Ethics.

[28] Bartholdson C, Lützén K, Blomgren K, Pergert P (2015). Experiences of ethical issues when caring for children with cancer. Cancer Nurs.

[29] Åström G, Jansson L, Norberg A, Hallberg IR (1993). Experienced nurses’ narratives of their being in ethically difficult care situations: The problem to act in accordance with one’s ethical reasoning and feelings. Cancer Nurs.

[30] Schaffer MA (2007). Ethical problems in end-of-life decisions for elderly Norwegians. Nurs Ethics.

[31] Norberg A, Norberg B, Gippert H, Bexell G (1980). Ethical conflicts in long-term care of the aged: nutritional problems and the patient-care worker relationship. Br Med J.

[32] Nordam A, Torjuul K, Sørlie V (2005). Ethical challenges in the care of older people and risk of being burned out among male nurses. J Clin Nurs.

[33] Wilmot S, Legg L, Barratt J (2002). Ethical issues in the feeding of patients suffering from dementia: a focus group study of hospital staff responses to conflicting principles. Nurs Ethics.

[34] Enes SPD, de VK (2004). A survey of ethical issues experienced by nurses caring for terminally ill elderly people. Nurs Ethics.

[35] DuVal G, Clarridge B, Gensler G, Danis M (2004). A national survey of US internists’ experiences with ethical dilemmas and ethics consultation. J Gen Intern Med.

[36] Murchison J. Ethnography Essentials. In: Designing, conducting, and presenting your research: Wiley; 2010.

[37] Venzon Cruz E, Higginbottom G (2013). The use of focused ethnography in nursing research. Nurse Res.

[38] Spradley JP (1979). The ethnographic interview.

[39] Atkinson P, Coffey A, Delamont S, Lofland J, Lofland L. Handbook of Ethnography: SAGE; 2001.

[40] Fetterman DM (2010). Ethnography: step-by-step.

[41] Bazeley P, Jackson K (2013). Qualitative data analysis with NVivo. Sec.

[42] Krippendorff K (2012). Content analysis: an introduction to its methodology.

[43] Lov om kommunale helse- og omsorgstjenester. (Healthcare law). 2016. https://lovdata.no/dokument/NL/lov/2011-06-24-30. Accessed 26 Oct 2016.

[44] Departementet för hälsa och välfärd. Lagrådsremiss: värdigt liv i äldreomsorgen. (Ministry of Health and Social Affairs. The council of legislation remittance: dignity life in elderly care]. Stockholm, Sweden; 2010. https://www.regeringen.se/rattsliga-dokument/proposition/2010/03/prop.-200910116/.

[45] Health Care Law. Stockholm, Sweden: Socialstyrelsen; 2006. https://www.socialstyrelsen.se/english.

[46] SFS 2001:453 Socialtjänstlagen. [The Social Service Act]. Stockholm, Sweden; 2001. http://www.riksdagen.se/sv/dokument-lagar/?doktyp=sfs&dokstat=g%c3%a4llande+sfs. Accessed 11 Nov 2016.

[47] Teeri S, Leino-Kilpi H, Välimäki M (2006). Long-term nursing Care of Elderly People: identifying ethically problematic experiences among patients, relatives and nurses in Finland. Nurs Ethics.

[48] Tønnessen S, Solvoll BA, Brinchmann BS (2016). Ethical challenges related to next of kin - nursing staffs’ perspective. Nurs Ethics.

[49] Wagner N, Tabak N (1998). The old get equal care: myth or reality. Int J Nurs Pract.

[50] O’Brien M, Fiester A (2014). Who’s at the table? Moral obligations to equal-priority surrogates in clinical ethics consultations. J Clin Ethics.

[51] Rees J, King L, Schmitz K (2009). Nurses’ perceptions of ethical issues in the Care of Older People. Nurs Ethics.

[52] Rasoal D, Kihlgren A, James I, Svantesson M (2015). What healthcare teams find ethically difficult captured in 70 moral case deliberations. Nurs Ethics.

[53] Fischer Grönlund CE, Söderberg AI, Zingmark KM, Sandlund SM, Dahlqvist V (2015). Ethically difficult situations in hemodialysis care – nurses’ narratives. Nurs Ethics.

[54] Leuter C, Petrucci C, Mattei A, Tabassi G, Lancia L (2013). Ethical difficulties in nursing, educational needs and attitudes about using ethics resources. Nurs Ethics.

[55] Cohen J, Erickson J (2006). Ethical dilemmas and moral distress in oncology nursing practice. Clin J Oncol Nurs.

[56] Paola FA, Walker R (2006). Ethicians, ethicists and the goals of clinical ethics consultation. Intern Emerg Med.

[57] Jeffery R (1979). Normal rubbish: deviant patients in casualty departments. Sociol Health Illn.

[58] Dingwall R, Murray T (1983). Categorization in accident departments: “good” patients, “bad” patients and “children.”. Sociol Health Illn..

[59] Morgan S, Yoder LH (2012). A concept analysis of person-centered care. J Holist Nurs.

[60] Mc Cormack B, Mc Cance T (2016). Theory and Practice. Person-Centred Practice in Nursing and Health Care.

[61] Henricson M. Vetenskaplig teori och metod- från ide till examination inom omvårdnad. (Scientific theory and methodology- From idea to examination in Nurs Pract). Lund: Studentlitteratur; 2012.

[62] Gadamer H. Hermeneutics as theoretical and practical problem. 1979. https://www.jstor.org/stable/2184785?origin=crossref&seq=1/analyze. Accessed 8 Mar 2016.

[63] Lincoln YS, Guba EG., Naturalistic Inquiry, 1st edition. Newbury Park,California: SAGE Publications; 1985.

[64] Rasoal D, Skovdahl K, Gifford M, Kihlgren A. Clinical ethics support for healthcare personnel: an integrative literature review. HEC Forum. 2017. 10.1007/s10730-017-9325-4 PMC5688194 28600658

